# Thioredoxin Reductase-1 Inhibition Augments Endogenous Glutathione-Dependent Antioxidant Responses in Experimental Bronchopulmonary Dysplasia

**DOI:** 10.1155/2019/7945983

**Published:** 2019-01-21

**Authors:** Stephanie B. Wall, Rachael Wood, Katelyn Dunigan, Qian Li, Rui Li, Lynette K. Rogers, Trent E. Tipple

**Affiliations:** ^1^Neonatal Redox Biology Laboratory, University of Alabama at Birmingham, Birmingham, AL, USA; ^2^Division of Neonatology, University of Alabama at Birmingham, Birmingham, AL, USA; ^3^Center for Perinatal Research, The Research Institute at Nationwide Children's Hospital, Columbus, OH, USA

## Abstract

**Background:**

Aurothioglucose- (ATG-) mediated inhibition of thioredoxin reductase-1 (TXNRD1) improves alveolarization in experimental murine bronchopulmonary dysplasia (BPD). Glutathione (GSH) mediates susceptibility to neonatal and adult oxidative lung injury. We have previously shown that ATG attenuates hyperoxic lung injury and enhances glutathione- (GSH-) dependent antioxidant defenses in adult mice.

**Hypothesis:**

The present studies evaluated the effects of TXNRD1 inhibition on GSH-dependent antioxidant defenses in newborn mice *in vivo* and lung epithelia *in vitro*.

**Methods:**

Newborn mice received intraperitoneal ATG or saline prior to room air or 85% hyperoxia exposure. Glutamate-cysteine ligase (GCL) catalytic (Gclc) and modifier (Gclm) mRNA levels, total GSH levels, total GSH peroxidase (GPx) activity, and Gpx2 expression were determined in lung homogenates. *In vitro*, murine transformed club cells (mtCCs) were treated with the TXNRD1 inhibitor auranofin (AFN) or vehicle in the presence or absence of the GCL inhibitor buthionine sulfoximine (BSO).

**Results:**

*In vivo*, ATG enhanced hyperoxia-induced increases in Gclc mRNA levels, total GSH contents, and GPx activity. *In vitro*, AFN increased Gclm mRNA levels, intracellular and extracellular GSH levels, and GPx activity. BSO prevented AFN-induced increases in GSH levels.

**Conclusions:**

Our data are consistent with a model in which TXNRD1 inhibition augments hyperoxia-induced GSH-dependent antioxidant responses in neonatal mice. Discrepancies between *in vivo* and *in vitro* results highlight the need for methodologies that permit accurate assessments of the GSH system at the single-cell level.

## 1. Introduction

Bronchopulmonary dysplasia (BPD) is the most common respiratory morbidity of prematurity and is characterized by respiratory insufficiency. Although improvements have been made in the prevention and treatment of BPD, the pathophysiology is complex and involves activation of injury and repair pathways in developing lungs [1]. Infants with BPD have poor lung function due to inadequate alveolarization often resulting in lifelong decreases in pulmonary function [2]. Supplemental oxygen, while necessary to overcome ventilation-perfusion mismatch, contributes to the development of BPD by damaging delicate lung tissues [3]. Premature infants are particularly vulnerable to oxygen toxicity due to poorly developed antioxidant systems [4–6].

Thioredoxin reductase-1 (TXNRD1) reduces oxidized thioredoxin-1 (Trx1). Previous studies from our group have demonstrated that TXNRD1 inhibition protects against the deleterious effects of hyperoxic exposure [7–9]. Our data have consistently indicated that activation of nuclear factor erythroid 2-related factor 2 (Nrf2) is a primary mechanism of protection afforded by TXNRD1 inhibitors, which is consistent with other reports [10]. One cytoprotective pathway activated by Nrf2 promotes *de novo* synthesis of the antioxidant glutathione (GSH).

GSH is the most abundant low-molecular-weight antioxidant [11] and protects the lung from hyperoxic injury [12]. *De novo* GSH synthesis is controlled by the enzymes GSH synthetase and *γ*-glutamate cysteine ligase (GCL). GCL is a heterodimer consisting of catalytic (Gclc) and modifier (Gclm) subunits. GCL is the rate-limiting step in *de novo* GSH synthesis [13, 14]. Once produced, GSH serves many important intracellular and extracellular roles in the lung including acting as a cofactor for enzymatic antioxidant pathways in cells and epithelial lining fluid, scavenging free radicals, protecting against oxidative species, aiding metabolism of xenobiotics, and regulating inflammation [15]. Intracellularly, most GSH are either free or protein-bound. Free GSH, under normal conditions, is mostly reduced. Under highly oxidizing conditions, two GSH molecules become disulfide linked resulting in the formation of GSSG. GSSG can accumulate within tissues, and the ratio of GSH to GSSG is commonly employed as an index of redox status. Previous reports have established associations between decreased plasma and tracheal aspirate GSH/GSSG ratios and BPD incidence in human infants [16]. The majority of GSH-mediated reductive reactions involve catalysis. GSH peroxidases (GPx), of which there are at least 8 known isoforms, are selenoenzymes, so named due to the presence of an active site selenocysteine (Sec) residue. GPx catalyzes the reduction of peroxides by GSH resulting in the formation of GSSG. GPX2 is an inducible isoform that may be protective in settings of oxidative stress such as cigarette smoke exposure [17]. Furthermore, hyperoxia-induced increases in GPX2 are Nrf2-dependent in the murine lung [18].

Our group identified TXNRD1 inhibition as a novel strategy to prevent lung injury. Administration of the TXNRD1 inhibitor aurothioglucose (ATG) is protective in murine models of acute lung injury (ALI), and protection is associated with increases in GSH-dependent antioxidant defenses [7, 19]. More recently, our group demonstrated that ATG inhibited pulmonary TXNRD1 activity and significantly attenuated hyperoxic lung injury in a neonatal mouse model of BPD [9]. Given these collective findings, the present studies tested the hypothesis that ATG-mediated attenuation of murine neonatal hyperoxic lung injury correlates with enhanced GSH-dependent antioxidant defenses.

## 2. Methods

### 2.1. Animal Model

All mouse work was performed using protocols approved by the University of Alabama at Birmingham IACUC. Four time-dated pregnant C3H/HeN mice (Envigo) were allowed to undergo a natural delivery, and pups of both sexes from all litters were randomly mixed and equally distributed between dams. Within 12 h of birth, pups received either 25 mg/kg aurothioglucose (ATG) or saline (SA) intraperitoneally (i.p.). Pups were then exposed to either room air (RA, 21% O_2_) or hyperoxia (85% O_2_) for 24, 48, or 168 h which corresponds to postnatal days (d) 1, 3, and 7, respectively, with the day of birth being 0 d. We previously reported the effects of hyperoxia and ATG on lung and body weights at day 7 in this model [9]. Hyperoxic exposures were performed in a custom-made plexiglass chamber with O_2_ controlled using a BioSpherix ProOx P110 controller (Parish, NY). To avoid hyperoxic mortality, dams were rotated out of hyperoxia with a paired room air dam daily. Upon completion of exposure, pups were euthanized with ketamine/xylazine (200/20 mg/kg, i.p.) and lungs were collected within 5 min of euthanasia. Tissues were flash frozen in liquid nitrogen. For GSH assays, lungs were cardiac perfused with 10 ml of PBS before collection. Tissues were stored at −80°C until assessments were performed.

### 2.2. Cell Culture

Murine (mouse) transformed club cells (mtCCs, Dr. Francisco Demayo (NIH)) were maintained in DMEM supplemented with 10% FBS and 50 U/ml penicillin/streptomycin (Gibco). Cells were plated at equal densities and were treated at ~80-90% confluence with 0.5 *μ*M AFN (Sigma) or vehicle control (dimethyl sulfoxide, DMSO, Fisher) for up to 24 hr. Buthionine sulfoximine (BSO) was dissolved in sterile phosphate-buffered saline and used at a final concentration of 22.5 *μ*M in cell media.

### 2.3. Sample Preparation

Approximately, 10 *μ*g of tissue was homogenized (on ice, Dounce homogenizer) in 100 *μ*l of lysis buffer consisting of 10 mM Tris buffer (pH 7.4), containing 0.1% Triton X-100 and 100 *μ*M diethylenetriamine pentaacetic acid (DTPA) with protease inhibitors (Thermo Scientific). For cell culture experiments, cells were washed once in PBS and were lysed in the buffer described above. Tissue and cell lysates were centrifuged at 20,000 × *g* for 10 min, and supernatant was collected. Protein concentrations were determined by the bicinchoninic acid (BCA) assay (Pierce).

### 2.4. Western Blot

Samples were loaded onto 4-20% Criterion or Mini-PROTEAN TGX gels (Bio-Rad), transferred to PVDF membranes (Trans-Blot, Bio-Rad), blocked with 5% milk in Tris-buffered saline containing 0.05% Tween 20 (TBST), and probed with rabbit anti-GPX2 (ab137431, Abcam; 1 : 1000 in 5% milk in TBST) followed by a goat anti-rabbit IgG-HRP secondary antibody (sc-2004; Santa Cruz Biotechnology; 1 : 5000 in TBST). Membranes were developed using Clarity ECL Substrate (Bio-Rad) and imaged using a ChemiDoc System (Bio-Rad). For loading control, membranes were reprobed with a mouse anti-*β*-actin antibody (sc-47778, Santa Cruz; 1 : 1000 in milk in TBST) followed by an anti-mouse IgG-HRP secondary antibody (HAF007; R&D Systems; 1 : 5000 in TBST). Pixel density of GPX2 was normalized to the respective actin band density.

### 2.5. Quantitative Real-Time Polymerase Chain Reaction (qRT-PCR)

Purified RNA (RNeasy and QIAcube; Qiagen, Valencia, CA) was reverse transcribed to cDNA using a PrimeScript RT Master Mix (Takara/Clontech, Mountain View, CA). PCR was performed using a Premix Ex Taq probe (Takara/Clontech) and Rotor-Gene Q instrument (Qiagen) with the primers for 18S (Hs99999901_s1), murine Gclc (Mm00802655_m1), and murine Gclm (Mm00514996_m1) (Applied Biosystems/Thermo Fisher; Foster City, CA). Cycle threshold (ΔCT) values were normalized to 18S, and then, fold changes were calculated relative to saline/room air (RA) using 2^−ΔΔCT^.

### 2.6. GSH Recycling Assay

Total glutathione (expressed as GSH equivalents: 2GSH + GSSG) levels were assessed in cellular lysates by the Tietze recycling assay [20]. Oxidized glutathione (GSSG) was measured by reacting samples with 2-vinylpyridine for 1 h. The reaction was then terminated with triethanolamine (TEA). 3 to 10 *μ*g of tissue homogenate or cell lysate was loaded into a 96-well plate; no more than 20 *μ*g was loaded for GSSG. Absorbance was monitored at 412 nm over 2 minutes in the presence of 0.5 mM dithionitrobenzene (DNTB), 0.24 mM NADPH, and GSH reductase (1 : 3000) in ammonium sulfate (G3664, Sigma). Standard curves were produced for both GSH and GSSG. Linear regression analysis was used to calculate GSH concentrations that were then normalized to the protein concentration.

### 2.7. GSH Peroxidase Activity

GPx activity in lung homogenates (~4-8 *μ*g) and cells (~50-70 *μ*g) was measured via the hydrogen peroxide-dependent consumption of NADPH at 340 nm over 5 minutes in the presence of 0.2 mM NADPH, 1.5 mM reduced GSH, 1.0 mM sodium azide, 0.5 mM DTPA, GSH reductase (1 : 427, Sigma), and 1.6 mM hydrogen peroxide (Fisher) in 50 mM Tris-HCl buffer at pH 7.0.

### 2.8. Statistics

Data (expressed as mean ± SEM) were tested for homogeneity of variances, log-transformed when indicated, and analyzed by 2-way ANOVA (exposure and treatment as independent variables) followed by Tukey's multiple comparison tests using GraphPad Prism® 7.0. Significance was accepted at *p* < 0.05.

## 3. Results

### 3.1. ATG Enhances GCLC but Not GCLM mRNA Levels in the Lungs of Hyperoxia-Exposed Newborn Mice

**Figure 1:**
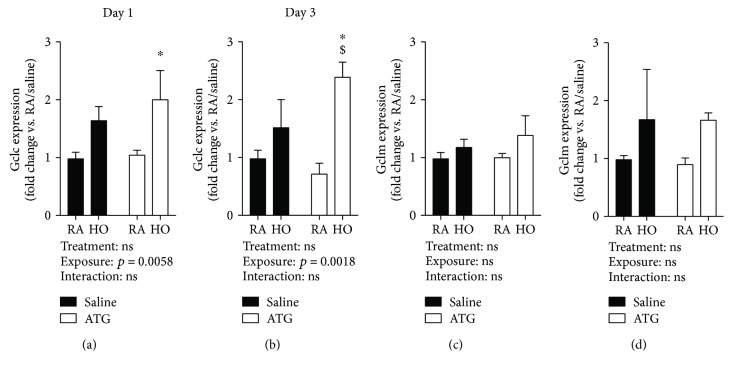
Lung Gclc and Gclm mRNA levels. Pups were dosed with saline or 25 mg/kg ATG and exposed to room air (RA) or 85% hyperoxia (HO) for 1 day (a, c) or 3 days (b, d) (*n* = 3-6). Data are expressed as fold change relative to saline/RA. ^∗^*p* < 0.05 vs. RA/saline; ^$^*p* < 0.01 vs. RA/ATG.

ATG inhibits TXNRD1 in the lungs of hyperoxia-exposed mice [9]. In an adult model of ALI, the protective effects of TXNRD1 inhibition correlated with increased Gclm mRNA levels [7]. Gclc and Gclm expression levels were measured in the lungs from day 1 and 3 pups to test the effects of hyperoxia and ATG on newborn lungs. Two-way ANOVA indicated an independent effect of hyperoxia on Gclc both at day 1 and day 3 (Figure 1). Post hoc analyses indicated significant increases in Gclc levels in the lungs from ATG/hyperoxia mice when compared to saline/RA controls at both 1 d (1.0 ± 0.1 vs. 2.0 ± 0.5-fold, *p* = 0.0456) (Figure 1(a)) and 3 d (1.0 ± 0.1 vs. 2.4 ± 0.2-fold, *p* = 0.02) (Figure 1(b)). There were no effects of hyperoxia or ATG on Gclm mRNA levels at either time point (Figures 1(c) and 1(d)).

### 3.2. Effects of ATG on Lung GSH Levels

**Figure 2:**
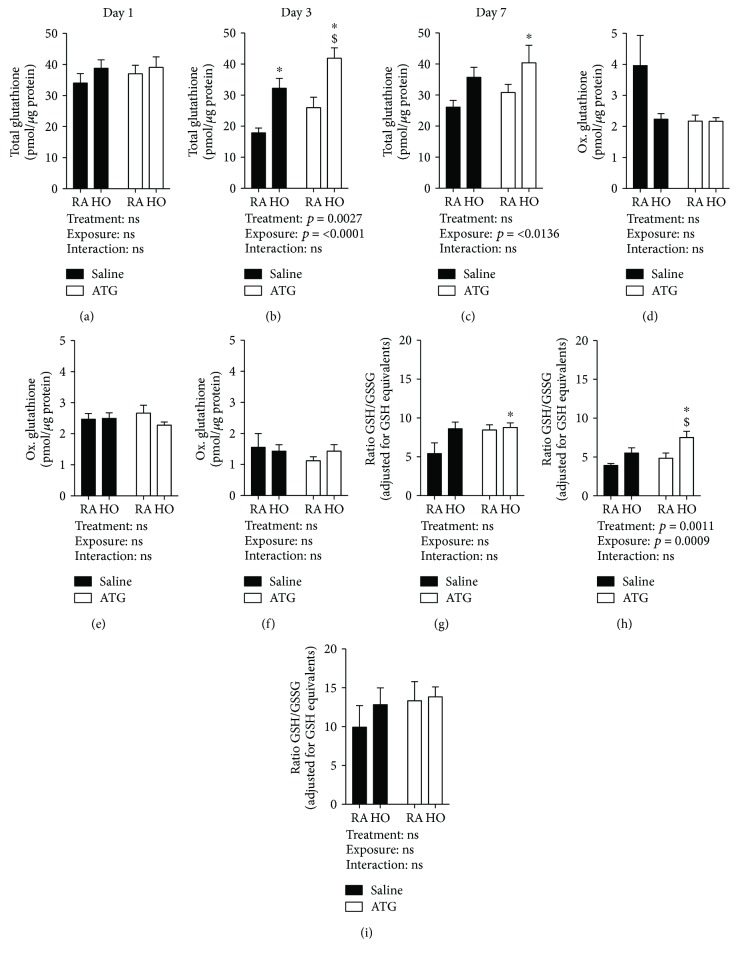
Lung glutathione levels. Pups were dosed with either saline or 25 mg/kg ATG and exposed to room air (RA) or hyperoxia (HO) for up to 7 days (*n* = 4-6). Total (a–c) and oxidized glutathione levels were determined (d, e) as described, and ratios of total to oxidized glutathione (adjusted for reduced glutathione equivalents) were calculated (g–i). ^∗^*p* < 0.05 vs. saline/RA; ^$^*p* < 0.05 vs. ATG/RA.

ATG-mediated attenuation of lung injury in adult murine models is associated with enhanced lung GSH levels, and protection is GSH-dependent [7, 19]. To determine the effects of hyperoxia and ATG on lung GSH levels in our murine BPD model, tissue GSH and GSSG levels were measured following exposure and treatment as outlined above. At day 1, there were no effects of hyperoxia or ATG on total GSH levels (Figure 2(a)). By day 3, two-way ANOVA indicated independent effects of both hyperoxia and ATG (Figure 2(b)). GSH levels were significantly greater in the lungs from saline/hyperoxia mice (32.6 ± 3 pmol/*μ*g) than from saline/RA controls (18.25 ± 1 pmol/*μ*g, *p* = 0.005). Compared to saline/RA controls, total GSH levels were significantly greater in the lungs from ATG/hyperoxia mice (42.3 ± 3 pmol/*μ*g, *p* < 0.001). Though GSH levels in the lungs from ATG/hyperoxia mice were greater than those in the lungs from hyperoxia/saline mice, the differences did not reach statistical significance (*p* = 0.07). At day 7, our analyses indicated only an independent effect of hyperoxia on lung GSH levels (Figure 2(c)). There were no effects of hyperoxia exposure or ATG on lung GSSG levels at any time point (Figures 2(d)–2(f)).

GSH/GSSG ratios, commonly employed as an index of redox balance, were calculated and analyzed. Day 3 was the only time point at which significant differences were identified (Figure 2(h)). Two-way ANOVA indicated effects of hyperoxia and ATG on GSH/GSSG ratios. Ratios were highest in the lungs from ATG/hyperoxia mice (7.7 ± 0.7), which were significantly different from those in the lungs from saline/RA (4.1 ± 0.1, *p* = 0.001) and ATG/RA (5.0 ± 0.5, *p* = 0.01) mice. Calculated GSH/GSSG ratios were greater than those in the lungs from ATG/hyperoxia mice when compared to saline/hyperoxia mice (5.7 ± 0.5); however, the differences did not reach statistical difference (*p* = 0.06).

### 3.3. Hyperoxia Enhances Lung GPx Protein Expression and Activity

**Figure 3:**
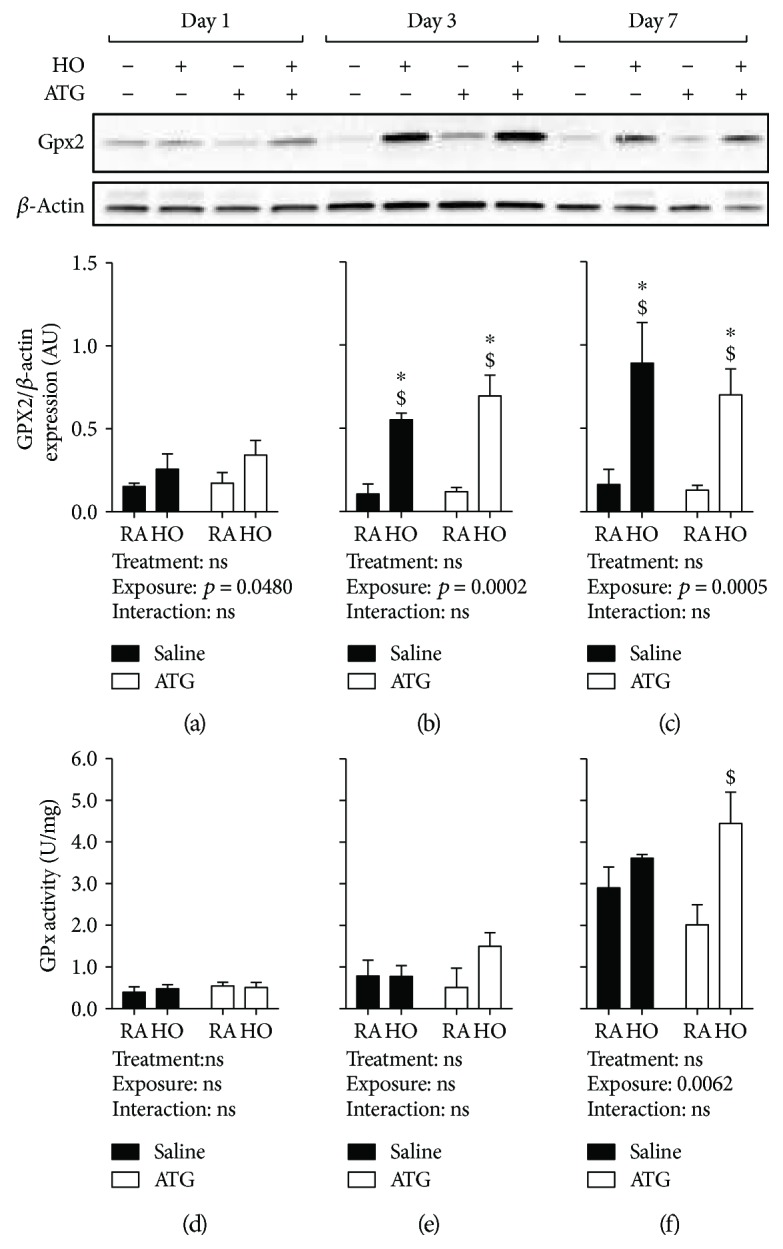
Lung glutathione peroxidase-2 expression and total GPx activity. Pups were dosed with either saline or 25 mg/kg ATG and exposed to either room air (RA) or hyperoxia (HO) for up to 7 days (*n* = 3-5). (a–c) Relative GPX2 density normalized to *β*-actin loading control. Total GPx activity (d–f). ^∗^*p* < 0.05 vs. saline/RA; ^$^*p* < 0.05 vs. ATG/RA.

GPX2 is an oxidative stress-inducible GPx isoform in the lung [17]. Thus, we assessed GPX2 protein expression in the lungs from RA- or hyperoxia-exposed neonatal mice treated with saline or ATG. GPX2 expression was not different between groups after day 1 (Figure 3(a)). At day 3, however, two-way ANOVA indicated an independent effect of hyperoxia (Figure 3(b)). Specifically, GPX2 protein levels were approximately 5 times greater in both saline/hyperoxia and ATG/hyperoxia lungs when compared to saline/RA lungs. An independent effect of hyperoxia was also identified at 7 d (Figure 3(c)), and GPX2 protein levels remained 5 times greater in both saline groups.

We cannot exclude the presence of other GPx isoforms in the lung. Nevertheless, measurements of GPx protein expression alone do not directly correlate with total GPx activity. We therefore determined GPx activity in lung homogenates from all 4 groups at days 1, 3, and 7 (Figures 3(d)–3(f)). Developmentally, total lung GPx activity is greatest at day 7 (Figure 3(f)). Though there were no independent effects of hyperoxia or ATG on lung GPx activity at day 1 or 3, our data indicated an independent effect of hyperoxia on GPx activity at day 7 (Figure 3(f)). At this time point, absolute GPx activity was greatest in the lungs from ATG/hyperoxia mice.

### 3.4. TXNRD1 Inhibition Enhances Gclm mRNA Levels in Murine Lung Epithelial Cells

**Figure 4:**
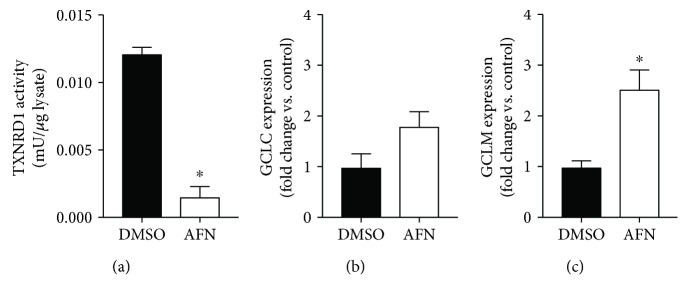
TXNRD1 activity and GCLC and GCLM expression in murine transformed club cells. mtCCs were treated with 0.5 *μ*M AFN or DMSO for 3 h (*n* = 3-5): (a) TXNRD1 activity and (b) GCLC and (c) GCLM mRNA expression. ^∗^*p* < 0.003.

Lung tissue is comprised of more than 40 cell types, and homogenates contain traces of blood or serum even when lungs are perfused prior to harvest. Thus, data derived from lung homogenates may not accurately reflect processes within airway epithelial cells, the cells in which TXNRD1 is primarily expressed [8]. We therefore evaluated the effects of TXNRD1 inhibition on GSH-dependent responses using murine transformed club cells (mtCCs), an SV-40 transformed mouse lung epithelial cell line commonly used by our group. Cells were continuously cultured in the presence or absence of the TXNRD1 inhibitor auranofin (AFN, 0.5 *μ*M). Consistent with our previous findings in mtCCs in which we used brief treatment (1 h) with a higher AFN concentration (1 *μ*M), TXNRD1 activity after 1 h of treatment was 87% lower in 0.5 *μ*M AFN-treated cells when compared to vehicle-treated controls (Figure 4(a)). After 3 h of continuous AFN treatment, Gclc mRNA levels were increased by 1.8-fold (*p* = 0.056) and GCLM mRNA levels were increased by 2.5-fold (Figure 4(b)) when compared to those of vehicle- (DMSO) treated cells.

**Figure 5:**
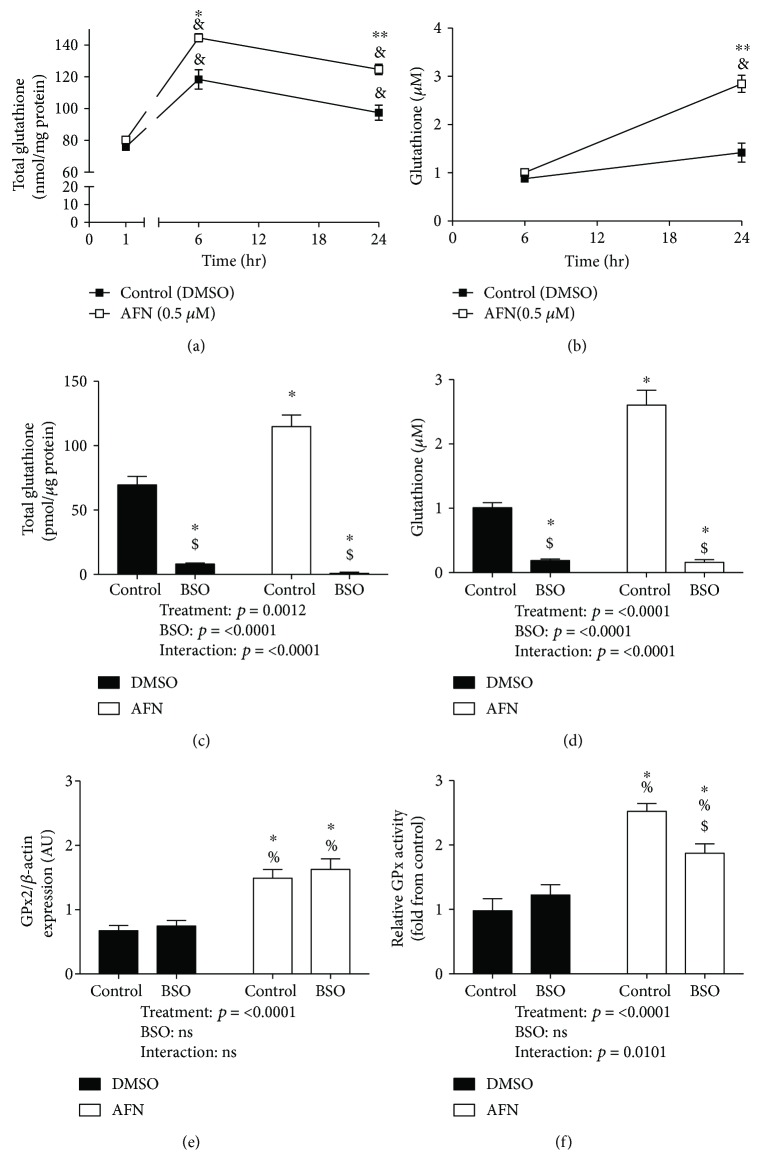
Total GSH levels and GPx activity in murine transformed club cells. mtCCs were treated with 0.5 *μ*M AFN (or vehicle, DMSO) for up to 24 h. Total GSH was measured at 1, 6, and 24 hr in the lysate (a) and at 6 and 24 hr in media (b). Data (*n* = 3-9) were analyzed by one-way ANOVA. In separate studies, mtCCs were treated with DMSO or 0.5 *μ*M AFN in DMSO in the presence or absence of buthionine sulfoximine (BSO). ^&^*p* < 0.05 vs. 1 h control; ^∗^*p* < 0.05 vs. 6 h control; ^∗∗^*p* < 0.05 vs. 24 h control. Total GSH levels in lysate (c) and media (d) at 24 hr were determined. GPX2 expression (e) and total GPx activity (f) were also determined. Expression data is representative of 5-6 samples from two independent experiments, and activity data is *n* = 3 per one independent experiment. Data (*n* = 3-6) were analyzed by two-way ANOVA followed by Tukey's post hoc analysis. ^∗^*p* < 0.05 vs. vehicle; ^$^*p* < 0.05 vs. AFN; ^%^*p* < 0.05 vs. BSO.

GSH levels were measured at 1, 6, and 24 h in cell lysates and at 6 and 24 h in media obtained from mtCCs continuously exposed to vehicle or 0.5 *μ*M AFN. In vehicle-treated control mtCCs, lysate GSH levels were increased by 157% at 6 h and 128% at 24 h when compared to levels at 1 h (Figure 5(a)). AFN treatment enhanced intracellular GSH levels by 122% at 6 h and 128% at 24 h when compared to respective vehicle-treated controls. Media GSH levels were not different between vehicle- and AFN-treated cells at 6 h (Figure 5(b)). In contrast, GSH levels were 2-fold greater in media from AFN-treated mtCCs at 24 h than from vehicle-treated controls.

To determine the impact of de novo GSH synthesis on changes in GSH contents, we utilized buthionine sulfoximine (BSO), a GCL inhibitor. mtCCs were cultured in the continuous presence or absence of 0.5 *μ*M AFN and/or 22.5 *μ*M BSO for 24 h. Thus, there were 4 experimental groups: vehicle, BSO, AFN, and BSO/AFN. At 24 h, our data revealed an independent effect of BSO on total GSH levels in cell lysate (Figure 5(c)) and media (Figure 5(d)). In lysates, BSO decreased total GSH levels to 13% of control values and 2.4% of control values in AFN-treated cells. In medium samples, BSO treatment in the absence or presence of AFN was associated with GSH concentrations that were approximately 20% compared to that of the DMSO-treated cells (Figure 5(d)).

In mtCCs treated with AFN for 24 h, we detected an independent effect of AFN on GPX2 protein levels (Figure 5(e)). Specifically, GPX2 expression was 2-fold greater in both AFN and BSO/AFN cells compared to vehicle or BSO groups alone. Similarly, our analyses indicated an independent effect of AFN on GPx activity in mtCCs. Compared to vehicle-treated controls, GPx activity was 2.5-fold greater in AFN-treated cells and 1.9-fold greater in BSO/AFN-treated cells than in controls (Figure 5(f)). GPx activity was 25% lower in BSO/AFN cells compared to cells cultured in the presence of AFN alone.

## 4. Discussion

GSH is the most abundant intracellular antioxidant and mediates susceptibility to and protection against neonatal and adult oxidative lung injury [11, 12, 15]. We previously demonstrated that ATG administered within 12 h of birth potently inhibits TXNRD1 and improves alveolarization in a murine BPD model [9]. The current manuscript extends our previous findings by demonstrating that TXNRD1 inhibition *in vivo* is associated with the following: (1) enhanced lung Gclc levels, increased total lung GSH levels, and elevated GSH/GSSG ratios in the lungs from hyperoxia-exposed mice at day 3 and (2) enhanced Gclm mRNA levels, increased intracellular and extracellular total GSH levels via enhanced de novo synthesis, and increased intracellular GPx activity in airway epithelial cells in vitro. Collectively, our data support a model in which TXNRD1 inhibition enhances pulmonary GSH-dependent antioxidant defenses promoting improved alveolarization.

In the current study, our data indicated that hyperoxia enhances *Gclc* but not *Gclm* expression in neonatal mouse lungs (Figure 1). Consistent with our findings with other Nrf2-regulated genes, the only significant increases in *Gclc* vs. control at d1 and d3 were detected in the ATG+hyperoxia group. This finding is suggestive of synergism between treatment and exposure on de novo GSH synthesis. Indeed, our data indicated independent effects of hyperoxia and ATG treatment on total lung GSH levels and GSH/GSSG ratios at d3 (Figures 2(b) and 2(e)). In absolute terms, the greatest levels of GSH and the highest GSH/GSSG ratios were found in the lungs from d3 ATG+hyperoxia mice.

Expression levels of both Gclc and Gclm are regulated by Nrf2. In our previous studies, we also identified a synergistic effect of ATG and hyperoxia on Nrf2-dependent pathways in the lungs from C3H/HeN mice [9]. One interpretation of these data is that TXNRD1 inhibition enhances Nrf2 dependent upon hyperoxic exposure resulting in proportionally greater induction of Nrf2-dependent pathways. The present data suggest that ATG enhances Nrf2-dependent de novo GSH synthesis by day 3. Importantly, day 3 immediately precedes initiation of the alveolar stage of development in the murine lung. Thus, we speculate that hyperoxia, in combination with ATG, enhances lung GSH levels to preserve proalveolarization pathways that are interrupted by hyperoxia in the absence of ATG.

An essential caveat to these findings is that global assessments of GSH and GSSG levels in whole lung homogenates preclude the identification of treatment and exposure effects on individual cell types. Thus, the magnitude of differences are not likely to specifically reflect cell-specific effects of hyperoxia and TXNRD1 inhibition on lung GSH or GSSG levels. It is possible that our data underestimate the degree of enhanced *de novo* GSH synthesis in individual cell types. Given that TXNRD1 is most abundantly expressed in airway epithelia in newborn mouse lungs, we speculate that ATG enhances Nrf2-dependent responses, including enhancement of GSH levels, in this compartment [8]. Airway epithelia are also the primary source for the production of epithelial lining fluid (ELF). Should ATG treatment promote the *de novo* synthesis of GSH in airway epithelia, as we found in our studies *in vitro* (Figure 5), it is likely that GSH levels in ELF would also be enhanced in hyperoxia-exposed mice. In adult mice, Nrf2-dependent GSH modulated innate immune responses to bacterial infection [21]. Unfortunately, currently available methods in our laboratory do not permit us to accurately determine GSH levels in small volumes such as those obtained by bronchoalveolar lavage of neonatal mouse lungs. Additional studies are underway to define cell type specificity of Nrf2-dependent gene induction in ATG-treated mice; however, such studies are beyond the scope of the present manuscript.

We chose to also assess GPX2 expression and GPx activity because recent studies have indicated that GPX2, which is regulated by Nrf2, mediates responses to lung injury [22]. GPX2 protein expression was increased by hyperoxia at all three time points (Figures 3(a)–3(c)); however, our analyses did not identify an independent effect of ATG. We next tested total GPx activity in lung homogenates from all groups (Figures 3(d)–3(f)). GPx activity was not significantly altered by ATG or hyperoxia until day 7. It is important to note that ATG is capable of inhibiting other selenocysteine-containing proteins including GPx [23]. Our data indicated that ATG, at the doses used in the present studies, did not inhibit GPx activity in neonatal murine lungs. These data are in contrast to TXNRD1 inhibition and are consistent with previous reports [9, 23–25]. GPX2 expression and total GPx enzymatic activity in whole lung homogenates from hyperoxia-exposed pups were unaffected by ATG pretreatment. Given the enzymatic capacity of GPx family proteins to catalyze GSH-dependent reduction of hydroperoxides, it is possible that additional upregulation was unnecessary to facilitate catalysis at d3, the day at which we saw maximal increases in total GSH levels and GSH/GSSG ratios.

We have consistently utilized mtCCs as a model system to study the effects of TXNRD1 inhibitors. Club cells provide ELF antioxidants, and a subpopulation serves as progenitor cells in lung repair [26, 27]. In contrast to our previous studies [8], we decreased the concentration of AFN to 0.5 *μ*M and continuously exposed cells to AFN or vehicle to more closely mimic our studies *in vivo*. Importantly, we did not observe evidence of AFN toxicity in the present studies which is in contrast to our previous studies with 1 *μ*M AFN (data not shown). TXNRD1 activity was inhibited by 87% in AFN-treated mtCCs in the present studies (Figure 4). AFN enhanced GCLM mRNA levels at 3 h; however, GCLC levels were not significantly different from each other (*p* = 0.058) by the statistical methods used.

In addition to measurements of intracellular GSH, we also sought to define the effects of TXNRD1 inhibition on extracellular GSH levels given the secretory property of club cells. Unsurprisingly, AFN enhanced total intracellular GSH levels by 6 h and differences were still present after 24 h of treatment (Figure 5(a)). Though not different at 6 h, AFN treatment also significantly enhanced GSH levels in the media at 24 h (Figure 5(b)). Extrapolation of these data suggest that TXNRD1 inhibition is likely to enhance ELF GSH levels which may, in part, contribute to its protective effects. Collectively, our data indicate a net increase of approximately 60% in total GSH contents (intracellular + extracellular) following TXNRD1 inhibition in mtCCs.

BSO alone significantly decreased intracellular GSH and medium GSH levels revealing the extent of basal GSH production under standard culture conditions (Figure 5(c)). The addition of BSO to AFN-treated cells prevented AFN-mediated increases in intracellular and extracellular GSH levels (Figures 5(c) and 5(d)). This indicates that AFN-mediated increases in GSH were mediated by de novo GSH synthesis. These and other studies indicate that *de novo* synthesis of GSH plays a major role in normal cell growth and proliferation [28, 29]. AFN treatment of mtCCs increased GPX2 levels by approximately 2-fold in the presence and absence of BSO (Figure 5(e)). GPx activity was also 2-fold greater following TXNRD1 inhibition in AFN-treated cells; however, concomitant BSO treatment significantly decreased GPx activity compared to AFN alone (Figure 5(f)). We interpret these findings to be reflective of cellular stress given observed morphological alterations in mtCCs concomitantly treated with AFN and BSO.

Therapeutic exogenous antioxidant administration has failed to prevent BPD [30]. Activation of endogenous antioxidant systems, such as that achieved by TXNRD1 inhibition, represents a novel approach to improving respiratory outcomes in premature infants. Our data suggest that augmentation of de novo GSH synthesis by therapeutic TXNRD1 inhibition could be a mechanism leading to improved respiratory outcomes in prematurely born infants. It should be noted that *de novo* GSH synthesis requires adequate levels of cysteine [31, 32]. In addition, selenium is required for selenocysteine synthesis and both TXNRD1 and GPx require selenocysteine for optimal enzymatic activity [33]. The majority of these nutrients are transplacentally acquired during the third trimester [34]. This means that extremely premature infants are inherently deficient and currently employed perinatal nutritional strategies are often insufficient to correct these deficiencies. Thus, we speculate that augmentation of GSH-dependent antioxidant defenses by TXNRD1 inhibitors is likely to be influenced by the bioavailability of both cysteine and selenium.

In conclusion, our data are consistent with a model in which TXNRD1 inhibition enhances Nrf2-dependent augmentation of the GSH antioxidant system, including *de novo* GSH synthesis, elicited by hyperoxic exposure in neonatal mice. In contrast to previous approaches that utilized exogenous antioxidant administration to prevent BPD, the use of therapeutic TXNRD1 inhibition enhances endogenous Nrf2-dependent responses and is associated with improved lung development. Our lab is currently defining the impact of neonatal nutritional deficiencies in cysteine and/or selenium on pulmonary Nrf2-dependent responses and GSH-dependent antioxidant defenses which will likely influence the translation of our findings from bench to bedside.

## Data Availability

No publicly available data were used for this manuscript.
